# Supplementary material for: Metabolomic Markers of Colorectal Tumor With Different Clinicopathological Features
Source: Front Oncol. 2020 Jun 17;10:981. doi: 10.3389/fonc.2020.00981 (PMC7311671; doi:10.3389/fonc.2020.00981)
Supplement: Supplementary file 1 [file Data_Sheet_1.PDF]

SUPPLEMENTARY MATERIAL FOR

Metabolomic markers of colorectal tumor tissues with different clinicopathological features

List of Supplementary Material

I. Supplementary Methods

1.1 Experimental protocol of metabolic profiling analysis for tissue by UPLC/Q-TOF-MS/MS

1.2 Data processing, multivariate and univariate analysis of metabolites, and identification of differential metabolites

II. Supplementary Figures

Supplementary Figure 1. Heatmap visualization constructed based on 373 differential variables.

Supplementary Figure 2. The Permutations Plot for OPLS-DA model based on the different clinical stage data set of tumor tissue.

Supplementary Figure 3. Heatmap visualization constructed based on 94 differential variables of the different clinical stage data set of tumor tissue.

Supplementary Figure 4. The Permutations Plot for OPLS-DA model based on the different pathologic stage data set of tumor tissue.

Supplementary Figure 5. Clinical stages at sample collection and the eventual metastasis of each individual metabolic cluster.

III. Supplementary Tables

Supplementary Table 1. Demographic and pathological characteristics of fifty-one colorectal cancer patients.

Supplementary Table 2. Colorectal cancer metabolites identified from the paired-tissue metabolomics study.

Supplementary Table 3. Pathway analysis of 373 positive and negative ion by Mummichog.

Supplementary Table 4. Fifty identifiable metabolites differential abundance between

early (I, II) and late-stage (III, IV) tumors.

Supplementary Table 5. Pathway analysis of 94 positive and negative ion by Mummichog

Supplementary Table 6. Twenty-six identifiable metabolites differential abundance between adenocarcinoma and non-adenocarcinoma tumors.

Supplementary Table 7. Pathway analysis of 43 positive and negative ion by Mummichog.

I. Supplementary Methods

1.1 Experimental protocol of metabolic profiling analysis for tissue by UPLC/Q-TOF-MS/MS

Chemicals and reagents Acetonitrile and methanol (HPLC grade) were purchased from Honeywell Burdick & Jackson (Muskegon, MI). Formic acid was purchased from Beijing Reagent Company (Beijing, China). All chemicals and regents were of HPLC grade available from commercial sources. Ultrapure water was prepared by an ultra-clear system (PURELAB Ultra; Veolia Water Solutions & Technologies, France).

Tissue sample preparation About 100 mg of tissue soaked in formaldehyde solution was placed in a mortar containing Liquid nitrogen and fully grounded. After the well-ground tissue was completely dissolved with 2 ml of methanol, all metabolites in the tissue were extracted and all proteins were precipitated. The solution obtained from step 1 was transferred to a 2 ml centrifuge tube, then was vortexed for 1 min; and centrifuged at 12,000 rpm for 10 min at 4°C. The supernatant was put in another 2 ml centrifuge tube and was dried with nitrogen. The substance dried by nitrogen in step 3 was dissolved with methanol (1:1) and the tissue soaking solution (300-400ul). Vortexed for 1 min and standing for 5 min, then centrifuged at 12,000 rpm for 10 min at 4°C. The supernatant was finally removed and transferred to auto sampler vial for metabolomics analysis by ULPLC/Q-TOF MSMS. A quality control sample (QC) was prepared by mixing aliquots from all supernatant samples (10 μL from each sample).

UPLC/Q-TOF-MS/MS analysis Chromatographic separation was performed on a 1.7-μm BEH C18 column [ACQUITY (HSS); Waters Corp., Milford, MA, USA; 2.1 mm × 100 mm] equipped with a UPLC system (ACQUITY UPLC; Waters Corp., USA). The temperatures of the column and autosampler were maintained at 35°C and 4°C, respectively. A sample (2 μl) of the preprocessed plasma was injected onto the column at a flow rate of 0.35 ml/min. The mobile phase consisted of water containing 0.1% formic acid (solution A) and acetonitrile (solution B). The elution gradient was

as follows: 2% B for 0.5 min; 2% to 20% B over 0.5 to 6.0 min; 20% to 35% B over 6.0 to 7.0 min; 35% to 70% B over 7.0 to 9.0 min; 70% to 98% B over 9.0 to 10.5 min; 98% B for 2.0 min; and then a return to 2% B for 6.0 min. Once the initial settings had been established, the column was equilibrated for 2.0 min. Acetonitrile was run every fifth sample as a blank solution and the plasma samples in the two analysis batches were injected alternately as five cases and five control samples.

Q-TOF MS/MS was performed with a mass spectrometer (Micromass Q-TOF mass spectrometer; Waters Corp., Manchester, UK) using an electrospray ionization (ESI) interface operated in both ion modes (ESI^- and ESI^+). The analytical parameters were as follows: capillary voltage, 2800 V in ESI^- or 3000 in ESI^+; sample cone voltage, 35 V; collision energy, 6 eV; source temperature, 110°C; desolvation gas (nitrogen) flow, 650 L/h; desolvation temperature, 320°C; cone gas (nitrogen) flow, 50 L/h; collision gas, argon; and MCP detector voltage, 2400 V. The Q-TOF mass acquisition rate was set at 0.4 s, with an interscan delay of 0.1 s. The scan mass range was from 50 to 1000 m/z. The data were collected in centroid mode, using the lock spray to ensure accuracy and reproducibility. A concentration of 200 pg/ml leucine–enkephalin was used as lock mass (m/z 554.2615) in ESI^- and (m/z 556.2771) in ESI^+. The lock spray frequency was set at 10 s, and the lock mass data were averaged over 10 scans for correction. The MS/MS spectra of metabolites were obtained by UPLC-MS/MS.

1.2 Data processing, multivariate and univariate analysis of metabolites, and identification of differential metabolites

Data processing The raw data were imported into MarkerLynx software incorporated in Masslynx software (version 4.1 SCN714). MarkerLynx ApexTrack peak integration was used for peak detection and alignment. The ApexTrack peak parameters were set as follows: peak width at 5% height, 1 s, and peak-to-peak baseline noise (calculated automatically). Collection parameters were set as follows: retention time (RT) range 0.5–10.5 min, mass range 50–1000 Da, mass tolerance, 0.05 Da; RT tolerance, 0.1 min; minimum intensity, 80; noise elimination level, 6.0; and deisotope data, yes. After being recognized and aligned, the intensity of each ion was

normalized to the summed total ion intensity of each chromatogram. The data-reduction process was handled in accordance with the “80% rule.”

Multivariate analysis of metabolites A matrix of samples against variables was generated and transferred to SIMCA-P version 13.0 software (Umetrics, Umeå, Sweden) for multivariate analysis. Principal component analysis (PCA) was first performed to check the outliers and the separation tendency. Orthogonal projections to latent structures discriminant analysis (OPLS-DA) was further applied to visualize the maximal difference between cases and controls. A default sevenfold (leave one-seventh of samples out) cross-validation procedure was used to assess the robustness of the models. Furthermore, permutation tests calculated by 100 randomizations were performed to avoid the overfitting of supervised OPLS-DA models. The variable importance in projection (VIP) values of all peaks from the OPLS-DA model was taken as a coefficient for peak selection.

Univariate analysis of metabolites The false detection rate (FDR)-corrected Mann-Whitney U tests was applied for the selection and validation of differential variables. The metabolites with statistical significance in both multivariate and univariate analyses (VIP >1.5 and P <0.05) were identified.

Differential metabolite identification Metabolite annotation was performed by comparing the exact m/z values and MS/MS spectra with those in free online databases Human Metabolome Database (HMDB, http://www.hmdb.ca/). If the potential MS/MS spectra were not available in online databases, the MassFragment application manager (MassLynx version 4.1, Waters) was applied to facilitate the MS/MS fragment ion analysis process via chemically intelligent peak-matching algorithms. Differential metabolites were finally confirmed using standard compounds based on both retention time and MS/MS spectra.

II. Supplementary Figure

Supplementary Figure Legends

Supplementary Figure 1. Heatmap visualization constructed based on 373 differential variables. Fifty-one pairs of tumor tissue and adjacent mucosa tissue were obtained from surgical resection of CRC patients,and subjected to UPLC/Q-TOF-MS/MS analyses. The identified differential metabolites were used to perform heatmap analyses. The color represents the metabolite concentration of each sample calculated by peak area normalization method. Note: Rows, samples; columns, differential variables. Color key indicates metabolite expression value: dark blue, lowest; dark red, highest.

Supplementary Figure 2. The Permutations Plot for OPLS-DA model based on the different clinical stage data set of tumor tissue. (A) Permutation test result of the OPLS-DA model in ESI^{-} model.; (B) Permutation test result of the OPLS-DA model in ESI^{+} model.

Supplementary Figure 3. Heatmap visualization constructed based on 94 differential variables of the different clinical stage data set of tumor tissue. Mann-Whitney U tests and OPLS-DA were used to identify metabolites significantly higher or lower in Stage III/IV tumors, compared to Stage I/II tumors (Benjamini-Hochberg corrected *P* value < 0.01, VIP>1.5). Metabolites were grouped, labeled on the left. Clinical stages at presentation were color-labeled. Note: Rows, samples; columns, differential variables. Color key indicates metabolite expression value: dark blue, lowest; dark red, highest.

Supplementary Figure 4. The Permutations Plot for OPLS-DA model based on the different pathologic stage data set of tumor tissue. (A) Permutation test result of the OPLS-DA model in ESI^{-} model.; (B) Permutation test result of the OPLS-DA model in ESI^{+} model.

Supplementary Figure 5. Clinical stages at sample collection and the eventual metastasis of each individual metabolic cluster. A rough estimate by a chi-square test indicated that there was no statistically significant difference among the three mClusters vs stages (*P*>0.05 Chi-Square).

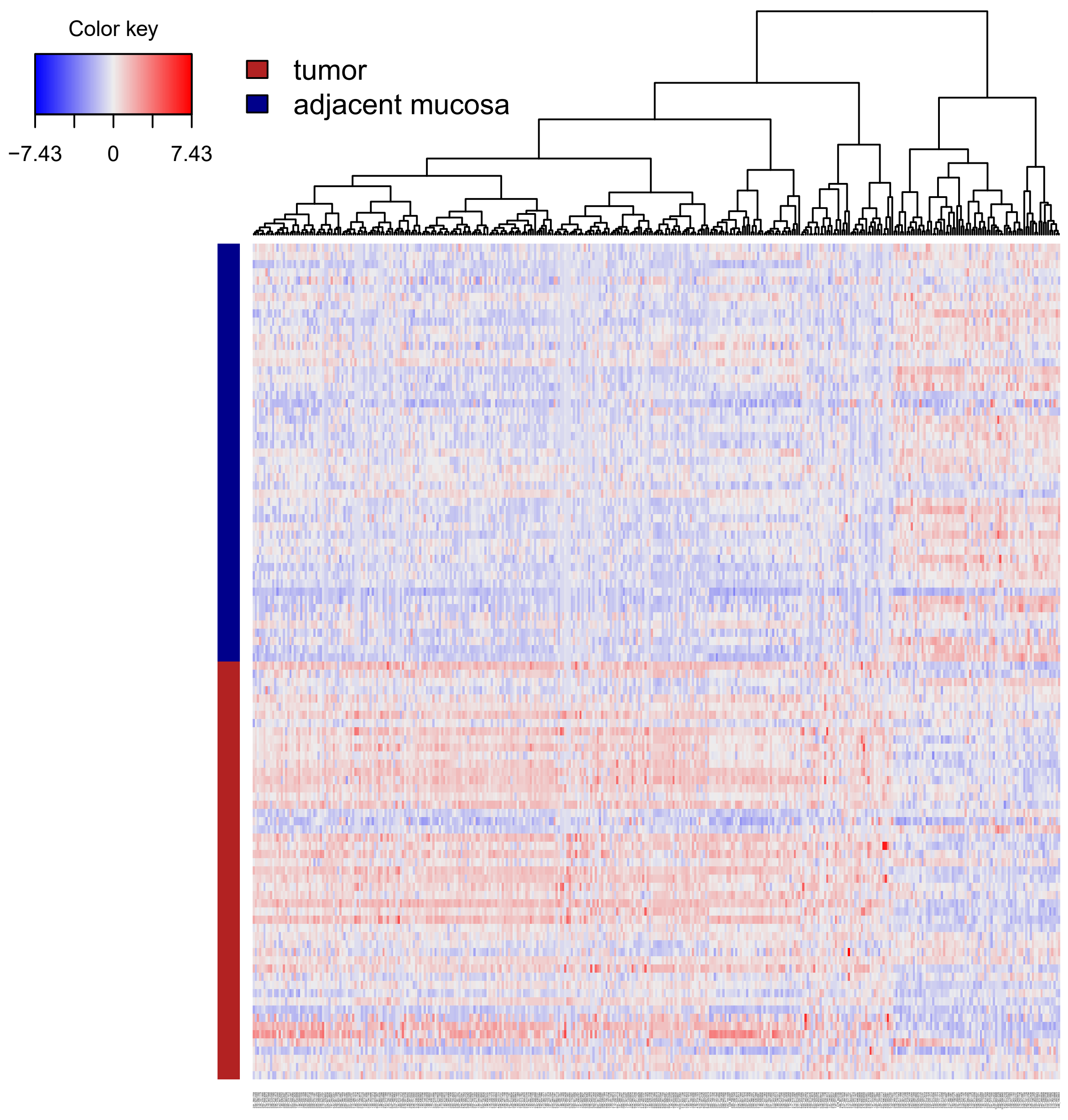

Supplementary Figure 1

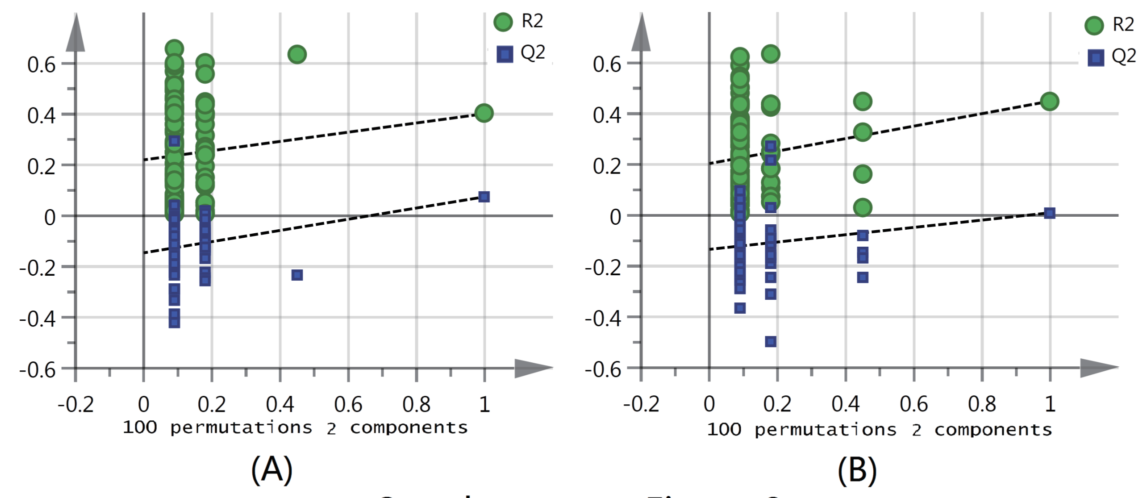

Supplementary Figure 2

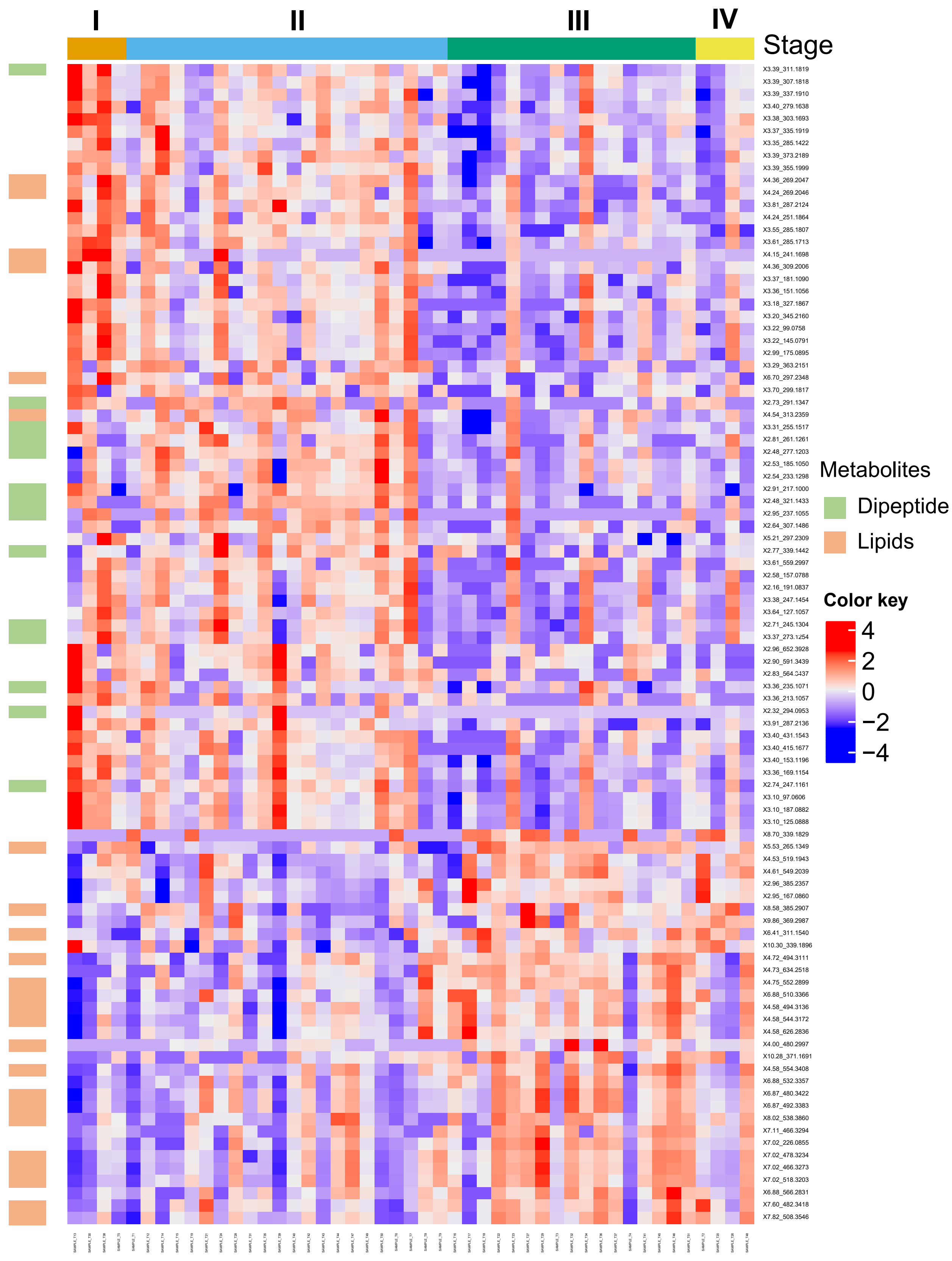

Supplementary Figure 3

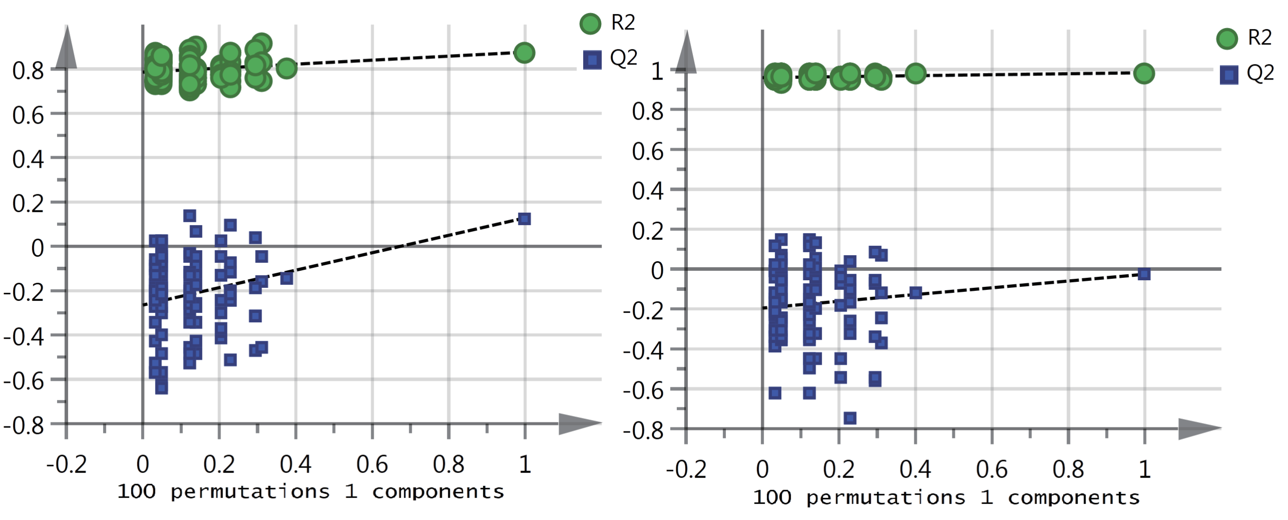

Supplementary Figure 4

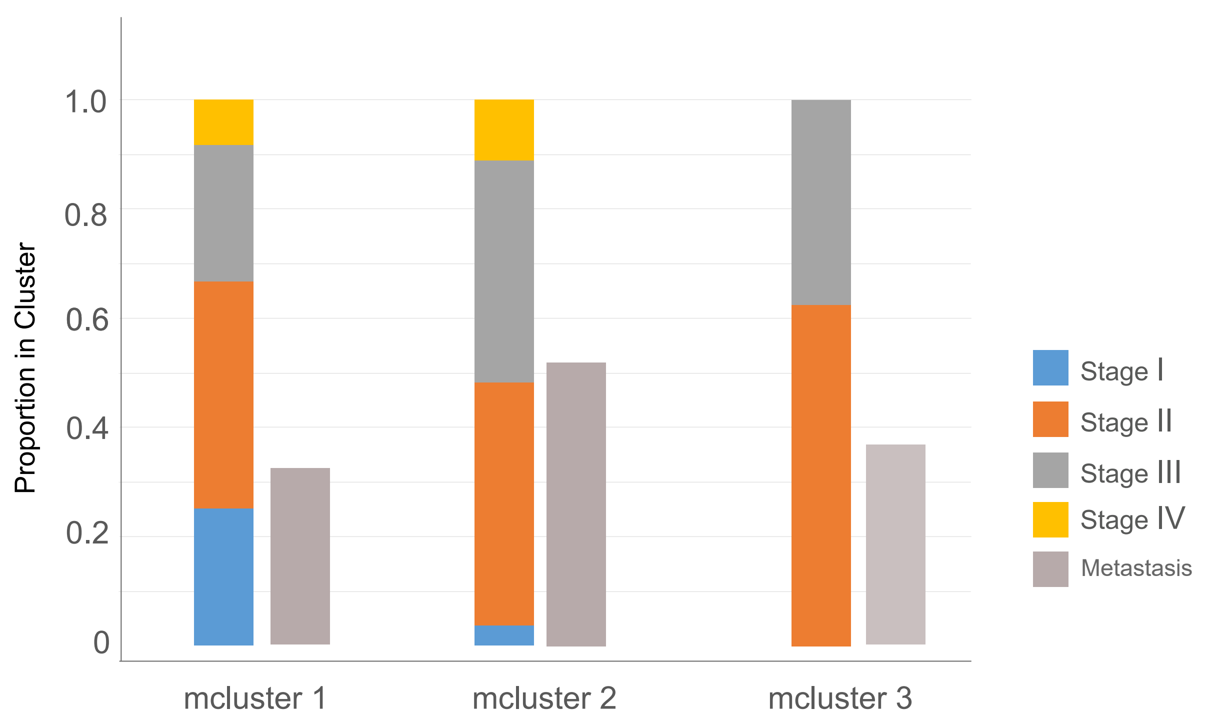

Supplementary Figure 5

III. Supplementary Table

Supplementary Table-1. Demographic and pathological characteristics of fifty-one colorectal cancer patients.

No.	Age	Gender	Location	Lymphatic metastasis	Distal metastasis	TNM staging	Histological type
1	65	Male	Spleen area	1	1	2	Non-adenocarcinoma
2	45	Male	Rectum	/	/	/	/
3	79	Male	Rectum	1	1	2	Adenocarcinoma
4	58	Female	Rectum	1	1	1	Adenocarcinoma
5	54	Male	Sigmoid colon	1	1	2	Non-adenocarcinoma
6	59	Male	Liver area	1	1	2	Non-adenocarcinoma
7	59	Female	Rectum	2	1	3	Adenocarcinoma
8	45	Male	Ascending colon	2	1	3	Adenocarcinoma
9	62	Female	Rectum	2	1	3	Non-adenocarcinoma
10	71	Female	Cecum	1	1	2	Non-adenocarcinoma
11	37	Male	Sigmoid colon	1	2	4	Adenocarcinoma
12	22	Female	Transverse colon	1	2	4	Non-adenocarcinoma
13	56	Male	Descending colon	1	1	2	Non-adenocarcinoma
14	43	Female	Rectum	2	1	3	Adenocarcinoma
15	49	Male	Rectum	2	1	3	Adenocarcinoma
16	39	Female	Cecum	1	1	2	Non-adenocarcinoma
17	35	Male	Rectum	/	/	/	/
18	71	Male	Liver area	1	2	4	Non-adenocarcinoma
19	56	Male	Rectum	2	1	3	Adenocarcinoma
20	47	Male	Descending colon	1	1	2	Non-adenocarcinoma

21	64	Male	Rectum	2	1	3	Non-adenocarcinoma
22	56	Male	Ascending colon	2	1	3	Non-adenocarcinoma
23	39	Female	Rectum	1	1	1	Non-adenocarcinoma
24	74	Female	Rectum	1	1	2	Adenocarcinoma
25	61	Female	Rectum	2	1	3	Non-adenocarcinoma
26	/	Female	/	/	/	/	/
27	56	Female	Sigmoid colon	2	1	3	Non-adenocarcinoma
28	50	Female	Sigmoid colon	1	1	2	Adenocarcinoma
29	68	Female	Ascending colon	2	1	3	Adenocarcinoma
30	56	Female	Rectum	2	1	3	Non-adenocarcinoma
31	70	Female	Rectum	1	1	1	Adenocarcinoma
32	67	Male	Sigmoid colon	1	1	2	Adenocarcinoma
33	71	Female	Sigmoid colon	2	1	3	Non-adenocarcinoma
34	51	Male	Rectum	1	1	2	Adenocarcinoma
35	56	Male	Rectum	2	1	3	Non-adenocarcinoma
36	55	Female	Spleen area	1	1	2	Adenocarcinoma
37	65	Male	Rectum	1	1	2	Adenocarcinoma
38	47	Male	Sigmoid colon	1	1	2	Adenocarcinoma
39	60	Male	Sigmoid colon	2	1	3	Adenocarcinoma
40	73	Female	Transverse colon	2	1	3	Adenocarcinoma
41	57	Female	Sigmoid colon	1	1	2	Adenocarcinoma
42	61	Male	Sigmoid colon	1	2	4	Adenocarcinoma
43	49	Female	Rectum	1	1	2	Adenocarcinoma
44	67	Female	Rectum	1	1	1	Adenocarcinoma
45	58	Male	Sigmoid colon	1	1	2	Adenocarcinoma
46	59	Male	Spleen area	2	1	3	Adenocarcinoma

47	/	/	/	/	/	/	/
48	72	Male	Rectum	1	1	2	Adenocarcinoma
49	55	Male	Rectum	1	1	2	Non-adenocarcinoma
50	54	Male	Rectum	1	1	2	Non-adenocarcinoma
51	74	Female	Spleen area	1	1	2	Non-adenocarcinoma

Note: Missing data: age ,2; sex, 2; tissue location, 2; T Stage, 4; M Stage, 4; N Stage, 4; TNM Stage, 4; Pathological type,4; histological_type, 4.

Supplementary Table 2. Colorectal cancer metabolites identified from the paired-tissue metabolomics study

NO.	RT[a]	Mass	HMDB number	Compounds	Adduct type	ESI mode	Tendency (Case/Control)
Nucleotide							
1	0.97	322.0385	HMDB0000095	Cytidine monophosphate	M-H	ESI-	↑
2	0.97	346.0424	HMDB0001397	Guanosine monophosphate	M+H-H2O	ESI+	↑
3	1	352.0486	HMDB0001202	Deoxycytidine monophosphate	M+FA-H	ESI-	↑
4	1.05	345.0069	HMDB0001554	Xanthylic acid	/	ESI+/ESI-	↑
5	1.14	346.05	HMDB0000045	Adenosine monophosphate	M-H	ESI-	↑
6	1.88	346.0483	HMDB0003540	Adenosine 3′-monophosphate	/	ESI+/ESI-	↑
7	1.88	374.0459	HMDB0000058	Cyclic adenosine monophosphate	/	ESI+/ESI-	↑
8	1.9	376.0606	HMDB0059612	7-Methylguanosine 5'-phosphate	/	ESI+/ESI-	↑
9	1.91	434.0699	HMDB0006880	Acetyl adenylate	M+FA-H	ESI-	↑
10	1.97	240.0907	HMDB0002224	5-Methyldeoxycytidine	M-H	ESI-	↑
11	2.01	376.0652	HMDB0000905	Deoxyadenosine monophosphate	M+FA-H	ESI-	↑

12	2.02	346.0572	HMDB0001044	2'-Deoxyguanosine 5'-monophosphate	/	ESI+/ESI-	↑
Amino acid							
13	0.7	198.0153	HMDB0006802	O-Phospho-4-hydroxy-L-threonine	M+H-H2O	ESI+	↑
14	2.09	273.1085	HMDB0000052	Argininosuccinic acid	M+H-H2O	ESI+	↑
15	2.54	233.1298	HMDB0000670	Homo-L-arginine	M+FA-H	ESI-	↓
16	3.3	187.0796	HMDB0000929	L-Tryptophan	M+H-H2O	ESI+	↑
Carbohydrate							
17	0.78	291.0708	HMDB0000497	5,6-Dihydrouridine	M+FA-H	ESI-	↑
18	0.78	403.0565	HMDB0001124	Trehalose 6-phosphate	/	ESI+/ESI-	↑
19	1.01	210.9931	HMDB0000618	D-Ribulose 5-phosphate	M-H20-H	ESI-	↑
20	1.28	181.0722	HMDB0000122	D-Glucose	M+H	ESI+	↑
21	1.28	210.9934	HMDB0001548	D-Ribose 5-phosphate	M-H20-H	ESI-	↑
22	1.82	403.0716	HMDB0006789	Lactose 6-phosphate	M-H20-H	ESI-	↑
23	1.89	210.9929	HMDB0000868	Xylulose 5-phosphate	M-H20-H	ESI-	↑
24	1.9	404.0552	HMDB0038413	2-Hydroxypentyl glucosinolate	M-H	ESI-	↑
25	1.94	358.0505	HMDB0000999	Phosphoribosylformylglycineamidine	M+FA-H	ESI-	↑
26	1.95	255.1035	HMDB0000855	Nicotinamide riboside	M+H	ESI+	↑
27	2.01	210.9933	HMDB0001489	Ribose 1-phosphate	M-H20-H	ESI-	↑
28	2.02	360.0788	HMDB0030934	Triglochinin	M+H	ESI+	↑
29	2.1	657.2433	HMDB0001081	(N-acetylneuraminosyl(a2-6)lactosamine)	M+H-H2O	ESI+	↑
Dipeptide							
30	0.97	231.1684	HMDB0028942	Leucyl-Valine	M+H	ESI+	↑

31	0.98	203.1008	HMDB0028688	Alanyl-Hydroxyproline	M+H	ESI+	↑
32	0.98	221.084	HMDB0028848	Glycyl-Phenylalanine	M-H	ESI-	↓
33	1	211.1107	HMDB0006695	Prolylhydroxyproline		ESI+	↑
34	1.87	286.1627	HMDB0013328	Pimelylcarnitine	M+H-H2<br>O	ESI+	↑
35	1.9	239.1036	HMDB0029105	Tyrosyl-Glycine	M+H	ESI+	↑
36	1.91	447.1004	HMDB0001117	4'-Phosphopantothenoylcysteine	M+FA-H	ESI-	↑
37	1.92	205.0828	HMDB0011667	gamma-Glutamylglycine	M+H	ESI+	↑
38	1.93	173.0382	HMDB0028684	Alanyl-Cysteine	M-H20-H	ESI-	↓
39	1.94	173.0497	HMDB0028753	Aspartyl-Glycine	M+H-H2<br>O	ESI+	↓
40	1.95	213.1007	HMDB0028885	Histidinyl-Glycine	M+H	ESI+	↑
41	1.95	284.1355	HMDB0028799	Glutaminylhistidine	M+H	ESI+	↑
42	1.99	199.1017	HMDB0029027	Prolyl-Threonine	M+H-H2<br>O	ESI+	↑
43	2.05	270.1091	HMDB0028705	Arginyl-Aspartic acid	M-H20-H	ESI-	↑
44	2.08	217.1034	HMDB0028778	Cysteinyl-Isoleucine	M+H-H2<br>O	ESI+	↓
45	2.1	234.1138	HMDB0028741	Asparaginyl-Threonine	M+H	ESI+	↑
46	2.14	270.1651	HMDB0028710	Arginyl-Hydroxyproline	M+H-H2<br>O	ESI+	↑
47	2.15	286.1076	HMDB0029072	Threoninyl-Tryptophan	M-H20-H	ESI-	↑
48	2.19	209.1019	HMDB0028689	Alanyl-Histidine	M+H-H2<br>O	ESI+	↑
49	2.21	233.1397	HMDB0028939	Leucyl-Threonine	M+H	ESI+	↑
50	2.23	213.0832	HMDB0028766	Aspartyl-Valine	M-H20-H	ESI-	↑

51	2.26	243.1246	HMDB0011177	Phenylalanylproline	/	ESI+/ESI-	↑
52	2.37	233.0643	HMDB0028774	Cysteinyl-Glutamate	M+H-H2O	ESI+	↓
53	2.47	299.1081	HMDB0028742	Asparaginyl-Tryptophan	M-H20-H	ESI-	↓
54	2.51	284.169	HMDB0028890	Histidinyl-Lysine	M+H	ESI+	↑
55	2.54	291.0946	HMDB0011741	gamma-Glutamyltyrosine	M-H20-H	ESI-	↓
56	2.56	312.1927	HMDB0028711	Arginyl-Histidine	M+H	ESI+	↑
57	2.57	269.0981	HMDB0028755	Aspartyl-Histidine	M-H	ESI-	↓
58	2.58	247.1105	HMDB0028986	Methionyl-Valine	M-H	ESI-	↓
59	2.58	257.1363	HMDB0029063	Threoninyl-Histidine	M+H	ESI+	↓
60	2.62	298.1548	HMDB0028918	Isoleucyl-Tryptophan	M-H20-H	ESI-	↑
61	2.7	267.1073	HMDB0028995	Phenylalanyl-Glycine	M+FA-H	ESI-	↓
62	2.74	242.1106	HMDB0028877	Hydroxyprolyl-Gamma-glutamate	M+H-H2O	ESI+	↓
63	2.74	247.0987	HMDB0028726	Asparaginyl-Asparagine	M+H	ESI+	↓
64	2.84	332.1255	HMDB0028830	Glutamyltryptophan	M-H	ESI-	↑
65	2.9	299.1348	HMDB0028898	Histidinyl-Valine	M+FA-H	ESI-	↓
66	2.94	346.1473	HMDB0029028	Prolyl-Tryptophan	M+FA-H	ESI-	↑
67	2.95	366.1659	HMDB0028716	Arginyl-Phenylalanine	M+FA-H	ESI-	↑
68	2.97	277.1514	HMDB0013243	Leucyl-phenylalanine	M-H	ESI-	↓
69	3.02	259.1429	HMDB0011171	gamma-Glutamylleucine	M-H	ESI-	↓
70	3.02	319.1678	HMDB0028965	Lysyl-Gamma-glutamate	M+FA-H	ESI-	↓
71	3.13	243.1332	HMDB0028928	Leucyl-Glutamate	M+H-H2O	ESI+	↑
72	3.28	225.1071	HMDB0028878	Histidinyl-Alanine	/	ESI+/ESI-	↓
73	3.28	243.1162	HMDB0011179	Prolylphenylalanine	M-H20-H	ESI-	↓

74	3.29	295.1187	HMDB0028980	Methionyl-Phenylalanine	M-H	ESI-	↓
75	3.3	249.0978	HMDB0029159	gamma-Glutamylthreonine	M+H	ESI+	↓
76	3.49	261.1268	HMDB0028996	Phenylalanyl-Hydroxyproline	M+H-H2 O	ESI+	↑
Lipid							
77	2.28	469.2104	HMDB0002421	7-Sulfocholic acid	M-H20-H	ESI-	↑
78	2.93	441.2492	HMDB0002504	3-Sulfodeoxycholic acid	M+H-H2 O	ESI+	↑
79	3.18	415.2179	HMDB0002886	6-Keto-prostaglandin F1a	M+FA-H	ESI-	↓
80	3.29	363.2151	HMDB0001337	Leukotriene A4	M+FA-H	ESI-	↓
81	3.43	467.258	HMDB0010321	3,17-Androstanediol glucuronide	M-H	ESI-	↑
82	3.47	423.2035	HMDB0000418	18-Hydroxycortisol	M+FA-H	ESI-	↓
83	3.48	387.2142	HMDB0002277	2,3-Dinor-6-keto-prostaglandin F1 a	M+FA-H	ESI-	↓
84	3.49	397.2233	HMDB0001335	Prostaglandin I2	M+FA-H	ESI-	↑
85	3.57	481.2605	HMDB0010351	11-beta-Hydroxyandrosterone-3-glucuroni de	M-H	ESI-	↑
86	3.64	355.2401	HMDB0004239	13,14-Dihydro PGF2a	M-H	ESI-	↑
87	3.7	379.235	HMDB0002082	Bisnorcholic acid	M-H	ESI-	↑
88	3.72	319.2149	HMDB0002265	14,15-DiHETrE	M-H20-H	ESI-	↓
89	3.78	325.2191	HMDB0010213	17 HDoHE	M-H20-H	ESI-	↓
90	3.8	381.2458	HMDB0001085	Leukotriene B4	M+FA-H	ESI-	↑
91	3.86	367.218	HMDB0000385	12a-Hydroxy-3-oxocholadienic acid	M-H20-H	ESI-	↑
92	3.92	381.2487	HMDB0012518	11'-Carboxy-gamma-tocotrienol	M-H20-H	ESI-	↑
93	3.95	313.2299	HMDB0000315	16-A-Hydroxypregnenolone	M-H20-H	ESI-	↓
94	4	480.2997	HMDB0000874	Tauroursodeoxycholic acid	M-H20-H	ESI-	↑
95	4.02	313.2299	HMDB0000363	17a-Hydroxypregnenolone	M-H20-H	ESI-	↓

96	4.09	409.24	HMDB0003259	Dihydrocortisol	M+FA-H	ESI-	↑
97	4.1	201.1136	HMDB0000792	Sebacic acid	M-H	ESI-	↓
98	4.12	703.3375	HMDB0036338	25-Acetyl-6,7-didehydrofevicordin F 3-glucoside	M-H	ESI-	↑
99	4.14	411.2367	HMDB0000949	Tetrahydrocortisol	M+FA-H	ESI-	↑
100	4.26	315.2439	HMDB0000253	Pregnenolone	M-H	ESI-	↓
101	4.39	315.2432	HMDB0011532	MG(0:0/15:0/0:0)	M-H	ESI-	↓
102	4.4	373.2056	HMDB0007003	CPA(16:0/0:0)	M-H20-H	ESI-	↓
103	4.42	438.2101	HMDB0012639	20-Hydroxy-leukotriene E4	M+H-H2 O	ESI+	↑
104	4.54	353.2269	HMDB0001220	Prostaglandin E2	M+H	ESI+	↓
105	4.69	295.2176	HMDB0004667	13S-hydroxyoctadecadienoic acid	M-H	ESI-	↑
106	4.69	613.4435	HMDB0071165	TG(10:0/13:0/8:0)	M+FA-H	ESI-	↑
107	4.74	830.6059	HMDB0000593	PC(18:1(9Z)/18:1(9Z))	M+FA-H	ESI-	↑
108	4.76	219.1323	HMDB0036389	3-Phenylpropyl isovalerate	M-H	ESI-	↑
109	4.93	345.2443	HMDB0011531	MG(0:0/14:1(9Z)/0:0)	M+FA-H	ESI-	↑
110	5.05	345.2381	HMDB0011562	MG(14:1(9Z)/0:0/0:0)	M+FA-H	ESI-	↑
111	5.18	293.2031	HMDB0004668	13-OxoODE	M-H	ESI-	↑
112	5.35	800.5935	HMDB0007952	PC(15:0/22:1(13Z))	M-H	ESI-	↑
113	5.42	267.2247	HMDB0030997	Cyclohexaneundecanoic acid	M-H	ESI-	↑
114	5.43	271.2193	HMDB0010734	(R)-3-Hydroxy-hexadecanoic acid	M-H	ESI-	↓
115	5.65	323.2506	HMDB0029797	(Z)-15-Oxo-11-eicosenoic acid	M-H	ESI-	↑
116	5.73	305.216	HMDB0013156	16-alpha-Hydroxyandrosterone	M-H	ESI-	↑
117	5.75	317.2058	HMDB0010202	12-HEPE	M-H	ESI-	↑
118	5.88	299.2485	HMDB0061661	9-hydroxyoctadecanoic acid	M-H	ESI-	↓
119	6.02	496.3347	HMDB0010382	LysoPC(16:0)	M+H	ESI+	↑

120	6.63	217.1328	HMDB0000888	Undecanedioic acid	M+H	ESI+	↓
121	6.93	546.3512	HMDB0010393	LysoPC(20:3(5Z,8Z,11Z))	M+H	ESI+	↑
122	7	301.2106	HMDB0001999	Eicosapentaenoic acid	M-H	ESI-	↑
123	7.92	355.2823	HMDB0011538	MG(0:0/18:2(9Z,12Z)/0:0)	M+H	ESI+	↓
124	7.93	395.2756	HMDB0012530	11-Hydroxyeicosatetraenoate glyceryl ester	M+H	ESI+	↓
125	7.93	414.3224	HMDB0013333	3-Hydroxy-9-hexadecenoylcarnitine	M+H	ESI+	↓
126	8.03	283.2352	HMDB0011530	MG(0:0/14:0/0:0)	M-H20-H	ESI-	↑
127	8.04	395.2216	HMDB0002664	Prostaglandin E3	M+FA-H	ESI-	↑
128	8.23	253.2086	HMDB0003229	Palmitoleic acid	M-H	ESI-	↑
129	8.23	529.4244	HMDB0092933	DG(8:0/18:0/0:0)	M+FA-H	ESI-	↑
130	8.4	253.2095	HMDB0031053	(E)-6-Hexadecenoic acid	M-H	ESI-	↑
131	8.53	205.1884	HMDB0040377	3-Pentadecenal	M-H20-H	ESI-	↑
132	8.53	303.2213	HMDB0001043	Arachidonic acid	/	ESI+/ESI-	↑
133	8.53	361.1857	HMDB0000319	18-Hydroxycorticosterone	M-H	ESI-	↑
134	8.54	629.4581	HMDB0000674	PA(16:0/16:0)	M-H20-H	ESI-	↑
135	8.61	361.1852	HMDB0000063	Cortisol	M-H	ESI-	↑
136	8.9	329.2418	HMDB0004708	9,12,13-TriHOME	M-H	ESI-	↑
137	8.91	559.4719	HMDB0007074	DG(15:0/18:2(9Z,12Z)/0:0)	M-H20-H	ESI-	↑
138	9.46	267.2256	HMDB0031046	9E-Heptadecenoic acid	M-H	ESI-	↑
139	9.48	329.2419	HMDB0004710	9,10,13-TriHOME	M-H	ESI-	↑
140	9.54	305.2399	HMDB0010378	5,8,11-Eicosatrienoic acid	M-H	ESI-	↑
141	9.54	529.4076	HMDB0006816	3-Hexaprenyl-4-hydroxybenzoic acid	M+H-H2O	ESI+	↓
142	9.55	633.4935	HMDB0007025	DG(14:0/20:4(5Z,8Z,11Z,14Z)/0:0)	M+FA-H	ESI-	↑
143	9.89	521.3729	HMDB0114757	LysoPA(24:1(15Z)/0:0)	M+H	ESI+	↓

144	10.05	331.2562	HMDB0002226	Adrenic acid	M-H	ESI-	↑
145	10.39	509.384	HMDB0035150	2'-Apo-beta-carotenal	M+H	ESI+	↓
Other							
146	1.02	137.0431	HMDB0000157	Hypoxanthine	M+H	ESI+	↑
147	1.02	146.0971	HMDB0003464	4-Guanidinobutanoic acid	M+H	ESI+	↑
148	1.06	249.0534	HMDB0001555	Pyridoxamine 5'-phosphate	M+H	ESI+	↑
149	1.14	256.1012	HMDB0002275	7,8-Dihydroneopterin	M+H	ESI+	↑
150	1.86	136.0602	HMDB0000034	Adenine	M+H	ESI+	↑
151	1.87	523.0658	HMDB0029212	Quercetin 3-O-glucuronide	M+FA-H	ESI-	↑
152	1.9	164.0564	HMDB0006037	8-Hydroxy-7-methylguanine	M+H-H2 O	ESI+	↑
153	1.92	449.1183	HMDB0029209	naringenin-7-O-glucuronide	M+H	ESI+	↑
154	1.97	271.0886	HMDB0000245	Porphobilinogen	M+FA-H	ESI-	↑
155	10.31	483.3668	HMDB0032111	Adlupone	M+H	ESI+	↓
156	2.1	180.0894	HMDB0011690	7-Aminomethyl-7-carbaguanine	M+H	ESI+	↑
157	2.12	148.0614	HMDB0001566	3-Methylguanine	M+H-H2 O	ESI+	↑
158	2.14	304.0941	HMDB0029839	Ascorbigen	M-H	ESI-	↑
159	2.31	241.064	HMDB0001857	1,3-Dimethyluric acid	M+FA-H	ESI-	↑
160	2.31	263.0971	HMDB0000235	Thiamine	M-H	ESI-	↑
161	2.35	414.1922	HMDB0010341	Dextrorphan O-glucuronide	M-H20-H	ESI-	↑
162	2.52	174.0921	HMDB0004225	2-Oxoarginine	M+H	ESI+	↑
163	2.75	127.0697	HMDB0003701	Dimethylbenzimidazole	M-H20-H	ESI-	↓
164	2.76	239.0907	HMDB0031517	(R)-3-Hydroxy-5-phenylpentanoic acid	M+FA-H	ESI-	↓
165	2.9	385.1649	HMDB0001091	3 Hydroxyquinine	M+FA-H	ESI-	↑
166	2.91	217.1	HMDB0000350	3-Hydroxysebacic acid	M-H	ESI-	↓

167	3.17	337.1363	HMDB0011687	Phenylbutyrylglutamine	M+FA-H	ESI-	↑
168	3.2	233.1314	HMDB0010725	(R)-3-Hydroxydecanoic acid	M+FA-H	ESI-	↓
169	3.6	167.1014	HMDB0010724	3-Oxodecanoic acid	M-H20-H	ESI-	↑
170	3.63	233.1454	HMDB0002203	3-Hydroxycapric acid	M+FA-H	ESI-	↑
171	4.69	195.1314	HMDB0010727	3-Oxododecanoic acid	M-H20-H	ESI-	↑
172	4.86	171.094	HMDB0004095	5-Methoxytryptamine	M-H20-H	ESI-	↑
173	8.53	259.2356	HMDB0033609	2- Pentadecylfuran	M-H20-H	ESI-	↑

[a]RT, retention time; Case/Control, case: tumor tissue, control: adjacent mucosa tissue; ↑, upgrade in tumor tissue; ↓, decline in tumor tissue.

Supplementary Table 3. Pathway analysis of 373 positive and negative ion by Mummichog

**Pathway**	**Overlap_size**	**Pathway_size**	***P*-value (raw)**	***P*-value**
**Negative mode (ESI-)**				
Linoleate metabolism	17	21	0.00012	0.005693
Leukotriene metabolism	31	50	0.000939	0.00573
Drug metabolism - cytochrome P450	27	48	0.013012	0.006287
Fatty acid activation	12	18	0.017944	0.006951
Prostaglandin formation from arachidonate	33	64	0.031624	0.006997
De novo fatty acid biosynthesis	11	17	0.031337	0.007887
Prostaglandin formation from dihomo gama-linoleic acid	5	6	0.038748	0.01118
Limonene and pinene degradation	5	6	0.038748	0.01118
Biopterin metabolism	9	15	0.088274	0.012531
Vitamin A (retinol) metabolism	18	36	0.131433	0.013277
Omega-3 fatty acid metabolism	5	7	0.091712	0.017255
Vitamin B5 - CoA biosynthesis from pantothenate	6	10	0.158468	0.023387

N-Glycan Degradation	4	6	0.172934	0.034215
Dynorphin metabolism	4	6	0.172934	0.034215
CoA Catabolism	4	6	0.172934	0.034215
Pentose phosphate pathway	13	29	0.343748	0.041055
**Positive mode (ESI+)**				
N-Glycan Degradation	6	8	0.017992	0.00075
Drug metabolism - cytochrome P450	22	51	0.07712	0.000995
Sialic acid metabolism	13	28	0.092295	0.001332
Heparan sulfate degradation	4	5	0.042719	0.001607
Vitamin B5 - CoA biosynthesis from pantothenate	5	8	0.082653	0.002154
Chondroitin sulfate degradation	3	3	0.035349	0.002221
Glycosphingolipid metabolism	11	25	0.162942	0.00271
Prostaglandin formation from dihomo gama-linoleic acid	4	6	0.09513	0.003208
Leukotriene metabolism	19	50	0.260187	0.004551
Omega-6 fatty acid metabolism	3	4	0.106692	0.005744
Vitamin H (biotin) metabolism	3	4	0.106692	0.005744
Tryptophan metabolism	22	60	0.304843	0.005954
Biopterin metabolism	6	14	0.296041	0.011921
Prostaglandin formation from arachidonate	22	63	0.408527	0.012283
Omega-3 fatty acid metabolism	4	8	0.248651	0.012868
Keratan sulfate degradation	4	8	0.248651	0.012868
Histidine metabolism	9	24	0.385836	0.016438
Butanoate metabolism	7	18	0.374535	0.018158
Glycerophospholipid metabolism	13	37	0.444616	0.019751
N-Glycan biosynthesis	5	12	0.354775	0.021228
Aminosugars metabolism	10	28	0.443216	0.022564

Aspartate and asparagine metabolism	17	50	0.485278	0.022807
De novo fatty acid biosynthesis	7	19	0.439384	0.027748
CoA Catabolism	3	6	0.310175	0.029356
Glycosphingolipid biosynthesis - globoseries	3	6	0.310175	0.029356
Glycolysis and Gluconeogenesis	7	20	0.503064	0.041215
Vitamin B1 (thiamin) metabolism	4	10	0.427937	0.041983
Glycosphingolipid biosynthesis - ganglioseries	4	10	0.427937	0.041983

Supplementary Table 4. Fifty identifiable metabolites differential abundance between early (I, II) and late-stage (III, IV) tumors.

NO.	RT[a]	Mass	HMDB number	Compounds	ESI mode	Adduct type
1	2.32	294.0953	HMDB0028743	Asparaginyl-Tyrosine	ESI-	M-H
2	2.48	321.1433	HMDB0033105	N2-Galacturonyl-L-lysine	ESI-	M-H
3	2.48	277.1203	HMDB0011176	L-phenylalanyl-L-hydroxyproline	ESI-	M-H
4	2.54	233.1298	HMDB0000670	Homo-L-arginine	ESI-	M+FA-H
5	2.71	245.1304	HMDB0029008	Phenylalanyl-Valine	ESI-	M-H-H2O
6	2.73	291.1347	HMDB0028887	Histidinyl-Histidine	ESI-	M-H
7	2.74	247.1161	HMDB0028986	Methionyl-Valine	ESI-	M-H
8	2.77	339.1442	HMDB0028919	Isoleucyl-Tyrosine	ESI-	M+FA-H
9	2.81	261.1261	HMDB0028913	Isoleucyl-Methionine	ESI-	M-H
10	2.91	217.1	HMDB0028694	Alanyl-Phenylalanine	ESI-	M-H-H2O
11	2.95	237.1055	HMDB0028895	Histidinyl-Threonine	ESI-	M-H-H2O

12	3.1	187.0882	HMDB0059783	3-Methylsubericacid	ESI-	M-H
13	3.2	345.216	HMDB0006219	13-cis-retinoic acid,Isotretinoin	ESI-	M+FA-H
14	3.29	363.2151	HMDB0010209	15-HEPE	ESI-	M+FA-H
15	3.35	285.1422	HMDB0000313	16b-Hydroxyestrone	ESI-	M-H
16	3.36	169.1154	HMDB0012183	8-Methylnonenoate	ESI-	M-H
17	3.36	235.1071	HMDB0028988	Phenylalanyl-Alanine	ESI-	M-H
18	3.37	273.1254	HMDB0011687	Phenylbutyrylglutamine	ESI-	M-H-H2O
19	3.39	311.1819	HMDB0028703	Arginyl-Arginine	ESI-	M-H-H2O
20	3.39	373.2189	HMDB0007003	CPA(16:0/0:0)	ESI-	M-H-H2O
21	3.55	285.1807	HMDB0000352	16alpha-Hydroxydehydroepiandrostero ne	ESI-	M-H-H2O
22	3.61	285.1713	HMDB0000309	3a,16b-Dihydroxyandrostenone	ESI-	M-H-H2O
23	3.81	287.2124	HMDB0000077	Dehydroepiandrosterone	ESI-	M-H
24	3.91	287.2136	HMDB0000899	5alpha-androstane-3,17-dione	ESI-	M-H
25	4	480.2997	HMDB0011130	PE(18:0/0:0)	ESI-	M-H
26	4.15	241.1698	HMDB0010730	3-oxo-tetradecanoic acid	ESI-	M-H
27	4.24	269.2046	HMDB0072845	MG(13:0/0:0/0:0)	ESI-	M-H-H2O
28	4.36	309.2006	HMDB0013623	9-oxo-12,13-epoxy-10-octadecenoic acid	ESI-	M-H
29	4.36	269.2047	HMDB0072861	MG(0:0/13:0/0:0)	ESI-	M-H-H2O
30	4.58	554.3408	HMDB0011511	PE(20:0/0:0)	ESI-	M+FA-H
31	4.58	494.3136	HMDB0006898	Chenodeoxyglycocholic acid	ESI-	M+FA-H
32	4.58	544.3172	HMDB0000951	Taurochenodeoxycholic acid	ESI-	M+FA-H
33	4.72	494.3111	HMDB0000708	Glycoursodeoxycholic acid	ESI-	M+FA-H
34	4.75	552.2899	HMDB0011499	LysoPE(0:0/24:6(6Z,9Z,12Z,15Z,18Z,2	ESI-	M-H

				1Z))		
35	5.21	297.2309	HMDB0010736	3-keto stearic acid	ESI-	M-H
36	5.53	265.1349	HMDB0000372	16-Oxoestrone	ESI-	M-H-H2O
37	6.41	311.154	HMDB0061027	2-hydroxyethinylestradiol	ESI-	M-H
38	6.7	297.2348	HMDB0059633	(9S,10S)-9,10-dihydroxyoctadecanoic acid	ESI-	M-H-H2O
39	3.31	255.1517	HMDB0028898	Histidinyl-Valine	ESI+	M+H
40	4.54	313.2359	HMDB0003871	13S-HpODE	ESI+	M+H
41	6.87	480.3422	HMDB0010407	PC(P-16:0/0:0)	ESI+	M+H
42	6.87	492.3383	HMDB0011481	LysoPE(0:0/20:0)	ESI+	M+H-H2O
43	6.88	510.3366	HMDB0011511	PE(20:0/0:0)	ESI+	M+H
44	7.02	518.3203	HMDB0010387	PC(18:3(6Z,9Z,12Z)/0:0)	ESI+	M+H
45	7.02	466.3273	HMDB0000138	Glycocholic acid	ESI+	M+H
46	7.02	478.3234	HMDB0010382	PC(16:0/0:0)	ESI+	M+H-H2O
47	7.6	482.3418	HMDB0011129	LysoPE(0:0/18:0)	ESI+	M+H
48	7.82	508.3546	HMDB0011482	LysoPE(0:0/20:1(11Z))	ESI+	M+H
49	8.02	538.386	HMDB0011490	LysoPE(0:0/22:0)	ESI+	M+H
50	8.58	385.2907	HMDB0011557	MG(0:0/22:6(4Z,7Z,10Z,13Z,16Z,19Z)/0:0)	ESI+	M+H-H2O

[a]RT, retention time

Supplementary Table 5. Pathway analysis of 94 positive and negative ion by Mummichog

Pathway	Overlap_size	Pathway_size	*P*-value (raw)	*P*-value
**Negative mode (ESI-)**				
Linoleate metabolism	7	21	0.073057	0.001555
Androgen and estrogen biosynthesis and metabolism	17	70	0.125102	0.001723
Vitamin A (retinol) metabolism	9	36	0.199974	0.003174
Omega-3 fatty acid metabolism	3	7	0.12024	0.003895
De novo fatty acid biosynthesis	5	17	0.186812	0.003948
C21-steroid hormone biosynthesis and metabolism	18	87	0.32218	0.004827
Drug metabolism - cytochrome P450	10	48	0.382566	0.007938
Urea cycle/amino group metabolism	9	46	0.474658	0.012985
Limonene and pinene degradation	2	6	0.303833	0.019796
Tryptophan metabolism	11	68	0.729774	0.041297
**Positive mode (ESI+)**				
Carnitine shuttle	5	25	0.003321	0.001247
Porphyrin metabolism	2	22	0.24149	0.010312
Bile acid biosynthesis	3	54	0.409312	0.013294
C21-steroid hormone biosynthesis and metabolism	4	83	0.479562	0.014328
Androgen and estrogen biosynthesis and metabolism	3	66	0.544338	0.021534
Vitamin E metabolism	2	36	0.461136	0.022977
Aspartate and asparagine metabolism	2	50	0.641177	0.040161
Leukotriene metabolism	2	50	0.641177	0.040161

Supplementary Table 6. Twenty-six identifiable metabolites differential abundance between adenocarcinoma and non-adenocarcinoma

tumors.

NO.	RT[a]	Mass	HMDB number	Compounds	ESI mode	Adduct type
1	0.97	298.0742	HMDB0000845	Neopterin	ESI-	M+FA-H
2	2.55	213.0178	HMDB0001351	Deoxyribose 1-phosphate	ESI-	M-H
3	3.36	347.2302	HMDB0000949	Tetrahydrocortisol	ESI-	M-H20-H
4	3.63	255.1085	HMDB0028795	Glutaminylglutamine	ESI-	M-H20-H
5	3.94	469.2467	HMDB0002421	7-Sulfocholic acid	ESI-	M-H20-H
6	4.12	301.2234	HMDB0002190	5,6-Epoxy-8,11,14-eicosatrienoic acid	ESI-	M-H20-H
7	4.22	353.2024	HMDB0012869	9'-Carboxy-gamma-tocotrienol	ESI-	M-H20-H
8	4.34	369.1847	HMDB0002759	Androsterone sulfate	ESI-	M-H
9	4.52	476.2744	HMDB0011477	LysoPE(0:0/18:2(9Z,12Z))	ESI-	M-H
10	5.3	516.3209	HMDB0010387	LysoPC(18:3(6Z,9Z,12Z))	ESI-	M-H
11	5.58	542.3401	HMDB0010395	LysoPC(20:4(5Z,8Z,11Z,14Z))	ESI-	M-H
12	5.58	624.3024	HMDB0001198	Leukotriene C4	ESI-	M-H
13	5.89	323.1734	HMDB0013243	Leucyl-phenylalanine	ESI-	M+FA-H
14	7.1	544.3543	HMDB0010393	LysoPC(20:3(5Z,8Z,11Z))	ESI-	M-H
15	7.1	554.3842	HMDB0011149	LysoPC(O-18:0)	ESI-	M+FA-H
16	7.57	271.2208	HMDB0010734	(R)-3-Hydroxy-hexadecanoic acid	ESI-	M-H
17	7.77	227.1938	HMDB0002221	2,6,10-Trimethylundecanoic acid	ESI-	M-H
18	9.12	241.2093	HMDB0032250	Dodecyl propionate	ESI-	M-H
19	10.1	255.2242	HMDB0031068	Isopalmitic acid	ESI-	M-H
20	10.31	345.2568	HMDB0010737	(R)-3-Hydroxy-Octadecanoic acid	ESI-	M+FA-H
21	10.33	567.3612	HMDB0114756	LysoPA(24:0/0:0)	ESI-	M+FA-H
22	10.34	511.3629	HMDB0002972	Vitamin K1 2,3-epoxide	ESI-	M+FA-H
23	2.3	316.1186	HMDB0028830	Glutamyltryptophan	ESI+	M+H-H2O

24	4.56	370.2865	HMDB0013329	trans-2-Tetradecenoylcarnitine	ESI+	M+H
25	6.45	263.2297	HMDB0000673	Linoleic acid	ESI+	M+H-H2O
26	6.52	285.1855	HMDB0003955	19-Hydroxyandrost-4-ene-3,17-dione	ESI+	M+H-H2O

Supplementary Table 7. Pathway analysis of 43 positive and negative ion by Mummichog

**Pathway**	**Overlap_size**	**Pathway_size**	***P*-value (raw)**	***P*-value**
Fatty acid activation	4	18	0.092743	2.18E-05
Fatty Acid Metabolism	3	12	0.106204	5.09E-05
De novo fatty acid biosynthesis	3	17	0.23038	0.000374
Carnitine shuttle	3	21	0.342279	0.001478
Vitamin E metabolism	4	34	0.434647	0.002318
Vitamin A (retinol) metabolism	4	36	0.480125	0.003702
Prostaglandin formation from arachidonate	6	64	0.615755	0.008622
Biopterin metabolism	2	15	0.443342	0.011149
Drug metabolism - cytochrome P450	4	48	0.712443	0.031625
Leukotriene metabolism	4	50	0.742804	0.041045
